# Dental Jurisprudence

**Published:** 1887-03

**Authors:** Richard Grady


ARTICLE IV.

### DENTAL JURISPRUDENCE.

BY RICHARD GRADY, D. D. S.,

Since the publication of my article on "Dental Jurisprudence, January, 1884, the following additional cases have come into my possession, which I now print as a supplement to the twelve cases therein noted. Several of these prove the importance of dentists keeping specific records of their dental work. My plea then that "Dental Jurisprudence should have not only a name but a local habitation" has been recognized, and there will be issued before June, 1887, "The American System of Dentistry" in three octavo volumes, volume III embracing *Dental Jurisprudence.*

RICHARD GRADY.
(Doctor of Dental Surgery.)

13. *Identification of the Prince Imperial.*—The occurrence of the Prince Imperial's death has revived a question which has been somewhat neglected by lawyers and physicians, viz: the importance of the teeth as a means of identification of deceased persons. The late Prince Imperial had been so much disfigured that identification would have been extremely difficult but that the Prince had had four small cavities in the first molar teeth filled with gold by Dr. Rottenstein, of Paris, and had met with a slight accident in April, 1876, from a blow on the front teeth, which had made it necessary to fill the teeth a little in order to smooth the enamel. These constituted signs which are unalterable even by ages; and, as careful dentists keep usually a record of such operations, as they afford a means of identification which is unerring, and which, as in the present instance, was of great value, and might, under certain circumstances, be of highest importance.

---

14. Miss Nellie D. Cooley, of Wilkesbarre, Pa. who disappeared in so mysterious a manner from her home on December 9, 1883, was found May 27, 1884, after a lapse of five months, in the Susquehanna River. The remains were so badly decomposed that all identification by general appearance was impossible, until Dr. C. S. Beck, dentist, of the above named place, was called as a witness by the coroner's jury, and he positively identified the remains by the structure of the teeth and the fillings he had inserted in some of them. So much for dental science.

15. "If you saw the man who bit that orange, would you be able to recognize him?" said an officer to me once. A robbery had been committed in a house, and the robber had bitten into a hard sour apple he found on the centre-table, and had then thrown it on the floor.

"Yes, I think I could," we replied; "and I believe I can so describe him to you that you can identify him. He has large upper central incisors, the front edge of the left one is turned out a little. The left lateral is gone and the right lateral is twisted nearly half way round. That man I believe was in my office a month since and had a little work done. Let me refer to my chart book.—His name is Mr. ———, and I filled three ———."

"But Doctor," interrupted the officer, "the young man you name is entirely above suspicion. It can't be he."

But it proved to be he, and he was sent to State's prison for the offence.

---

16. *The Teeth from a Medico-Legal Aspect.*—The identification of dead bodies and criminals is sometimes a matter of much perplexity. For instance; the features of a dead body may be distorted or destroyed; the clothes changed or unrecognizable; and no ordinary circumstances left to make identification clear. Some such a case occurred in Michigan. A man was found in a lake murdered. As the coroner was about dismissing the case as "unidentified," the neighboring dentist had the curiosity to look into the mouth. In a moment he said, "I have a chart of that mouth in my office," and though he could not then remember the name, he soon found it by referring to his chart book. It resulted in tracing the murderer,

---

17. *Twenty Years After. An Iowa Doctor discovers the remains of his Brother.*—A very remarkable case of the finding and identification of the remains of a Union soldier twenty years after he fell has just come to light. During the war a brother of Dr. Conoway, of Des Moines, Iowa, enlisted in a Pennsylvania regiment and went to the front. He was engaged in most of the battles of Virginia, and finally fell before Lynchburg. He was buried without being recognized, and appeared on the muster-roll after the battle as "missing." Young Conoway, so far as the family could learn was seen to fall in the front of a charge against the breastworks, and then all track of him was lost. The war passed by, and, despite the most careful inquires, no trace of the boy could be found. Last month Dr. Conoway attended a national medical convention in Pennsylvania, and when its sessions had closed, extended his journey into Virginia in search for the remains of his soldier brother. After visiting many battle fields, he finally came to Lynchburg and there discovered a man who had been a member of the same regiment as the deceased, and who had seen him fall. This young man had been a member of the burial party. Young Conoway had not been buried in the trenches, but in a separate ground on a hillock near by, which, the man said, he thought he could recognize. Adding him to the searching party, the battle ground was carefully scoured and the lone grave discovered. Of course the flesh had disappeared, but from a peculiarity of the teeth Dr. Conoway was fully able to identify the remains. Among the remnants of clothing was found a small phial tightly corked, inclosing a slip of paper, on which was written his brother's name.

---

18. *The St. Louis trunk Tragedy.*—The relatives of C. Arthur Preller, who was killed by Maxwell and his body packed in a trunk at the Southern Hotel, recently discovered a receipted bill among his effects by which they ascertained that Dr. Burnette, of San Francisco, had filled his teeth in March, 1878. They wrote to Dr. Burnette, who referred to his books, and then replied that he had filled teeth for Preller. The counsel for Mr. Preller's family in the suit they have brought to recover the insurance on his life, has written to Dr. Burnette to learn what work was done on

Preller's teeth. When he receives the information he intends to have the body exhumed and the teeth examined. It is believed the testimony thus secured will be very important, not only in the insurance case, but in the trial of Maxwell for murdering Preller.

---

19. *A Massachusetts Mystery. Detectives seeking for the Murderer of the Body found at Wrentham, Mass.*—The excitement over the finding of the skeleton of a woman in a field in Wrentham is still spreading in that neighborhood. The medical examiners have carefully looked over the skull and find that the ball passed completely through it, indicating that the shot was fired by some one who sat or stood at her right, and that by no means could she herself have used the weapon,

Last evening it was seen that the upper teeth were false, and that there was a small gold-filling used to give the set a genuine appearance. They were of superior workmanship, and were undoubtedly made by a city dentist.

---

20. *Miss May Hatch. The Identity of the Body.*—Miss May Hatch, of Baltimore, went to Norfolk, Va., June 16th, 1886. From there she took the steamer for Boston, and when on the ocean, it is claimed, committed suicide by drowning. To perfectly establish the identity of the remains of Miss Hatch, Dr. Norris, of North Charles street, the family dentist, examined the mouth and teeth of the body the morning before burial. The young lady had been at various times under his professional treatment. Comparison with diagrams in the doctor's possession left no room for doubt regarding the identity of the corpse.

---

21. Once more, at Paris, the impossible has happened. The case of Madame Menetret, the retired French milliner, who was mysteriously murdered at Villemonble, but whose assassin had long eluded the closest search of the police, has just evolved a new feature, which is surprisingly like that which, a generation ago, at Boston, identified Professor Webster as the slayer of Dr. Parkman. An artificial tooth has been found, the dentist who made it has sworn that he fitted it to the mouth of Madame Menetret, and the place where it was discovered and the attendant circumstances affix, with what looks terribly like certainty, the commission of the crime upon a woman named Euphrasie Mercier. This, it will be remembered, is precisely what occured in the Webster-Parkman case, and the counterpart to the incident which established the guilt of Webster, who was hung for the crime.

---

ARTICLE V.

—THE NINTH—

